# C-terminal frameshift mutations generate viable knockout mutants with developmental defects for three essential protein kinases

**DOI:** 10.1007/s42994-024-00165-5

**Published:** 2024-05-15

**Authors:** Yun Zhang, Miao-Miao Cui, Run-Nan Ke, Yue-Dan Chen, Kabin Xie

**Affiliations:** 1 National Key Laboratory of Crop Genetic Improvement, Hubei Hongshan Laboratory, Huazhong Agricultural University, Wuhan, 430070 China; 2 Hubei Key Laboratory of Plant Pathology, Huazhong Agricultural University, Wuhan, 430070 China

**Keywords:** CRISPR/Cas9, Lethal mutant, Protein kinases, Genome editing, C-terminus

## Abstract

Loss-of-function mutants are fundamental resources for gene function studies. However, it is difficult to generate viable and heritable knockout mutants for essential genes. Here, we show that targeted editing of the C-terminal sequence of the embryo lethal gene *MITOGEN-ACTIVATED PROTEIN KINASES 1* (*OsMPK1*) results in weak mutants. This C-terminal-edited osmpk1 mutants displayed severe developmental defects and altered disease resistance but generated tens of viable seeds that inherited the mutations. Using the same C-terminal editing approach, we also obtained viable mutants for a wall-associated protein kinase (Os07g0493200) and a leucine-rich repeat receptor-like protein kinase (Os01g0239700), while the null mutations of these genes were lethal. These data suggest that protein kinase activity could be reduced by introducing frameshift mutations adjacent to the C-terminus, which could generate valuable resources for gene function studies and tune protein kinase activity for signaling pathway engineering.

**Supplementary Information:**

The online version contains supplementary material available at 10.1007/s42994-024-00165-5.

Dear Editor,

Loss-of-function mutants are fundamental resources for gene function studies. However, it is difficult to generate viable and heritable knockout mutants for essential genes (Meinke et al. 2008). In the past decade, CRISPR/Cas9 genome editing has become a routine genetic tool for targeted gene knockout (Xie and Yang 2013; Zhu et al. 2020). Cas9 and guide RNAs (gRNAs) predominantly introduce 1 bp insertions/deletions (InDels) at targeting sites, via endogenous nonhomologous DNA end joining, during plant genome editing (Chen et al. 2022). Such frameshift mutations, within the protein coding regions, resulted in premature stop codons and thereby knocked out the function of the target gene. In practice, CRISPR/Cas9 is often recommended for the introduction of frameshift InDels adjacent to the start codon or within exons that encode conserved protein domains (Chen et al. 2021). Due to the high efficiency of CRISPR/Cas9 genome editing, viable seeds of null mutants of embryonically lethal genes are hard to generate through Cas9-mediated mutagenesis. For example, in our previous attempts to knock out rice mitogen-activated protein kinase (MAPK) genes (Xie et al. 2015), null mutants of two MAPK genes, *OsMPK1* (MSU ID: LOC_Os06g06090; RGP ID: Os06g0154500; also named *OsMAPK6*) and *OsMPK6* (MSU ID, LOC_Os10g38950; RGP ID, Os10g0533600), were found to generate lethal embryos in the T_0_ generation, but these mutants could not be preserved due to embryonic lethality (Minkenberg et al. 2017). During the construction of an arrayed CRISPR library of 1072 rice receptor-like kinase (RLK) and receptor-like cytoplasmic kinase (RLCK) genes (Chen et al. 2022), at least 20 knockout mutants of the RLK/RLCK genes displayed developmental defects and failed to generate viable seeds. These protein kinases are also important for rice immune signaling and abiotic stress responses (Dievart et al. 2020). The lack of stable knockout mutants of essential protein kinase genes hinders the dissection of signaling networks which control rice development, immunity, and abiotic stress tolerance.

Notably, Liu et al. (2015) obtained a *dwarf and small grain1* (*dsg1*) mutant in which the function of *OsMPK1* was knocked out*.* Interestingly, *dsg1* displayed developmental defects but generated very few viable seeds. The *dsg1* mutant carried a 1 bp deletion (referred to as − 1), which was located 54 bp upstream of the stop codon (Fig. 1A). This mutation generated a premature stop codon which deleted the last 19 amino acid residues of OsMPK1. As a result, the last α-helix (referred to as the αL16-helix in the MAPK structure (Canagarajah et al. 1997)) of the protein kinase domain is truncated in the *OsMPK1*^*dsg1*^ allele (Liu et al. 2015). In addition to those of *OsMPK1*, we recently reported that frameshift mutations that change the last 10–20 amino acid residues of two calcium-dependent protein kinase genes, *OsCPK18* and *OsCPK4*, altered Ca^2+^ sensitivities of both kinases and resulted in gain-of-function mutants that enhanced rice disease resistance and yield per plant (Li et al. 2022). On the basis of these findings, we hypothesized that frameshift mutations adjacent to the C-terminus of the kinase domain may generate functional knockout mutants or gain-of-function lines of essential protein kinase genes.

**Fig. 1:**
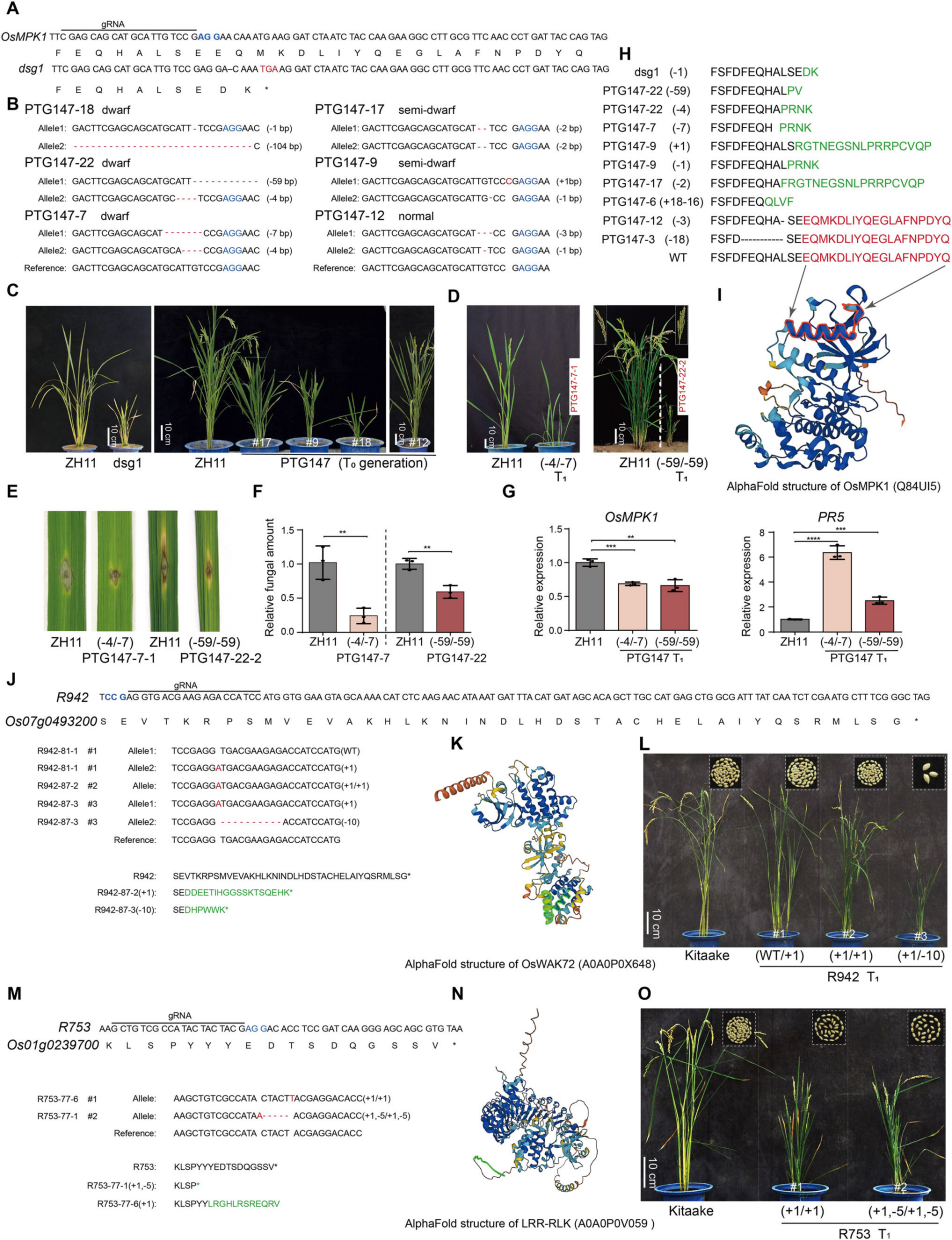
Targeted editing of the C-terminal sequences of *OsMPK1*, WAK *R942*, and the LRR-RLK *R753*. **A** Schematic illustrating the C-terminal coding sequences and corresponding protein sequences of *OsMPK1* and *dsg1*. The sequence under the line indicates the gRNA guide sequence, and the PAM sequence for CRISPR/Cas9 is shown in blue. Red letters and *, stop codon; −, deletion. **B** Representative genotype data of *OsMPK1* CRISPR lines (PTG147). These T_0_ lines were categorized into three types according to plant height (dwarf, semidwarf, normal). **C** and **D** Photos showing the heights of *dsg1* mutants and *OsMPK1*-edited plants (PTG147). The panicles of WT and PTG147-22-2 are shown in the left and right top corners, respectively, in **D**. Bar = 10 cm. **E** Comparisons of rice blast resistance of wild type plants (ZH11) and two *OsMPK1* edited plants (PTG147-22 and PTG147-7) at T_1_ generation. The photos were taken at 6 days after inoculation of *Magnaporthe oryzae* isolate 99-20-2. **F** The relative fungal amount in rice leaves inoculated with *M. oryzae*. The data are presented as the mean ± SD (*n* = 3 biological replicates). **G** Relative expression of *PR5* and *OsMPK1* in PTG147-7 and PTG147-22 lines at T_1_ generation. The data are presented as the mean ± SD (*n* = 3 biological replicates). ** and *** indicate *P* value < 0.01 and 0.001 (Student’s *t*-test), respectively. **H**–**I** Comparisons of C-terminal protein sequences of different *OsMPK1* alleles in PTG147 lines. The changes of protein sequences resulted from frameshift mutations are shown in green. Red letters indicate the last α-helix and C-terminal extension sequences of OsMPK1. The last α-helix is labeled with red outlines in AlphaFold protein structure (**I**). **J**–**O** Targeted editing of the C-terminal sequence of *R942* and *R753*. **J** and **M** Schematics showing gRNA positions in targeted genes. Representative genotype data for the *R942* and *R753* CRISPR editing lines are shown below the genome sequence. **K** and **N** AlphaFold predicted structure of R942 and R753. The accession number shown in brackets. The green outlines mark the last α-helix (**K**) and C-terminal extension sequences (**N**) in AlphaFold protein structures. **L** and **O** Photos of the CRISPR T_1_ lines *R942* (**L**) and *R753* (**O**) and viable seeds from one panicle (Kitaake, R942^WT/+1^, R942^+1/+1^) or whole plants (R942^+1/−10^, R753^+1/+1^, R753^(+1,−5)/(+1,−5)^), respectively. *WT* wild type; the number in brackets indicates the genotypes of target genes. Bar = 10 cm

To test the above hypothesis, we edited *OsMPK1* using a Cas9/gRNA whose cleavage site was 7 bp upstream of the deletion of *dsg1* (Fig. 1A). We obtained 18 positive T_0_ plants (referred to as PTG147) and genotyped them using Sanger sequencing (Table S1). These plants carried highly frequent biallelic frameshift mutations, ranging from 1 bp insertion (+ 1) to 141 bp deletion (− 141), at the Cas9 cleavage site (Fig. 1B). Interestingly, 11 *OsMPK1*-edited T_0_ plants exhibited moderate to extreme dwarfism and carried various frameshift mutations (Fig. 1B and C, Table S1; e.g., lines 18, 9, and 17), implying that changes in the C-terminal amino acid residues of OsMPK1 affect rice development to different extents. Furthermore, 7 T_0_ plants harboring monoallelic frameshift mutations exhibited normal morphology and seed development (e.g., PTG147-12; Fig. 1C). After self-fertilization, as we previously observed in O*sMPK1* CRISPR lines targeting 5′-end coding exons (Minkenberg et al. 2017), biallelic frameshift mutants were defective in embryogenesis and failed to develop seeds, except for 2 other lines (PTG147-22 and PTG147-7; Table S1). These two lines displayed similar dwarf phenotypes but generated very few viable seeds. The T_1_ plants of PTG147-22 and PTG147-7 also displayed an extremely dwarf phenotype and carried the desired homozygous mutations as did the parental T_0_ lines (Fig. 1D). We also tested rice blast resistance using a spot inoculation assay. The results showed that these two T_1_ lines had smaller lesions and less fungal growth than did the wild-type plants (Fig. 1 E and F). Consistently, the expression of *pathogenesis-related gene* 5 (*PR5)* was 2.5–6 times greater in comparison with that in the WT, while the expression of the *OsMPK1* transcript was slightly reduced (Fig. 1G), likely due to the positive feedback regulation in MAPK signaling (Schmidt et al. 2013). These data suggest that *OsMPK1* plays an essential role in development and negatively regulates rice blast resistance. The extremely dwarf T_1_ plant PTG147-22-2, which carried homozygous deletions of − 59/− 59, also produced several viable seeds (Fig. 1D). The phenotype of the PTG147-22 plants resembled that of the *dsg1* mutant, which has developmental defects and few viable seeds. Taken together, these data demonstrated that frameshift mutations close to the stop codon could generate heritable loss-of-function mutations in *OsMPK1*.

The null mutation of embryonic lethal and female/male sterile genes could be preserved as heterozygotes (Meinke et al. 2008). Indeed, 7 T_0_ lines of *OsMPK1*, which carry frameshift mutation in only one allele and WT sequence (or non-frameshift mutation) in the other allele, exhibited normal development. We show such example lines, PTG147-12 and PTG147-3, which carried − 3/− 1 and − 18**/**− 1 mutations in the T_0_ generation. As expected, segregated T_1_ plants with homozygous frameshift mutations (− 1/− 1) presented developmental defects and did not generate viable seeds. However, the heterozygous plants displayed normal development and seeding in the T_1_ generation, confirming that the null mutants of *OsMPK1* can be preserved as heterozygotes.

We further compared the protein sequences of the mutated *OsMPK1* alleles in these CRISPR/Cas9-edited plants (Fig. 1H and I). A total of 8 representative variants of mutated OsMPK1 proteins were obtained from the PTG147 plants. The variants were categorized into three types. Type I includes − 1, − 4, − 59, and − 7 bp, which result in a premature stop codon and truncate the last 21–23 amino acid residues of OsMPK1 as *dsg1*. Type II includes + 1 and − 2 bp mutations, which changed the last 20 and 22 amino acid residues, respectively. Type III includes − 3 and − 18 mutations, which did not change the reading frame but did delete 1 and 6 amino acid residues, respectively, in OsMPK1 (Fig. 1H). All frameshift mutations (Type I and Type II) in these plants resulted in deletion of the αL16 helix and the C-terminal extension sequence of OsMPK1. Because only the *OsMPK1*^*−59/−59*^ and *dsg1* mutations stably generated viable seeds, we hypothesized that the mutated proteins of *OsMPK1*^*−59/−59*^ may have leaky activity during rice embryogenesis.

Since the biochemical features of protein kinases are similar, we further assessed the effect of genome editing of C-terminal frameshift mutations in essential protein kinase genes through targeting the coding sequence close to the stop codon. To this end, we selected two rice protein kinases, a wall-associated protein kinase (WAK), Os07g0493200 (referred to as *R942*), and a leucine-rich repeat receptor-like protein kinase (LRR-RLK), Os01g0239700 (referred to as *R753*) (Fig. 1J–O). We previously obtained functional knockout T_0_ plants of both genes by editing the sequence close to the start codon, however none of them generated viable seeds (Chen et al. 2022). In this study, we edited the C-terminal sequences of *R942* and *R753* and obtained 2 and 4 T_0_ lines, respectively (Table S2). The T_0_ plants of both gene mutants displayed various phenotypes, as we observed in *PTG147* lines. We further analyzed the genotypes and phenotypes of the T_1_ progenies. For *R942*, plants with a homozygous 1 bp insertion (+ 1/ + 1) exhibited normal development, suggesting that this mutation did not affect gene function. Interestingly, the *R942* edited line with a heterozygous + 1/− 10 mutation exhibited dwarfism, implying that *R942*^*−10*^ was a null function allele. Again, the + 1 and − 10 mutations changed the last 39 amino acid residues of R942, which disrupted the C-terminal α-helix (Fig. 1J). For *R753*, two homozygous mutants with + 1/+ 1 and (+ 1, − 5)/(+ 1, − 5) genotypes were obtained. Both lines displayed strong developmental defects in the T_1_ generation but generated few viable seeds. Notably, the *R753* mutation locates in the C-terminal extension region, which is downstream of the last α-helix (Fig. 1N). These data imply that frameshift mutations close to the C-termini of protein kinases could generate mutants with a loss-of-function phenotype.

Protein kinases are fundamental components for cellular signaling transduction including many essential genes for plant embryo development and growth. By introducing frameshift mutations adjacent to the C-terminus, we generated mutants with strong developmental defects but could generate few viable seeds for three essential protein kinases. These data imply that amino acid residues in the last α-helix and C-terminal extensions are critical for kinase activity and editing these regions have a chance to generate functional defect mutant for essential protein kinases. These phenomena are consistent with the findings of MAPK studies in animals. For example, the α-L16-helix of p38α is involved in autophosphorylation-mediated activation of MAPK in animals (Diskin et al. 2007; Rothweiler et al. 2011). Substitution and phosphorylation of amino acid residues in this region changed MAPK activity in animal p38α and yeast Hog1 (Tesker et al. 2016). Our genome editing data of R942 and R753 imply that the C-terminal region of RLK is likely critical for kinase activities, as is the case for OsMPK1, but the biochemical mechanism of these phenomena requires further investigation. In summary, this study presents an approach for generating knockout mutants with viable seeds for essential protein kinase genes, which can potentially be engineered to tune protein kinase activities for crop improvement in the future.

## Materials and methods

### Plant materials and growth conditions.

Rice (*Oryza sativa* L. ssp. *Geng*) cv. Kitaake and Zhonghua 11 (ZH11) were used in this study. PTG147 lines were generated in the ZH11 background. R753 and R942 were generated from the cv. Kitaake background. For disease resistance evaluation, rice plants were grown in a greenhouse at 28 °C under 14 h day/23 °C and 10 h night conditions.

### Construction of CRISPR/Cas9 plasmids and rice transformation

For CRISPR/Cas9 genome editing, the targeting sites of *OsMPK1*, *R942* and *R753* were designed using CRISPR P 2.0. The genome editing plasmids were constructed as described previously (Chen et al. 2022). Briefly, for each gRNA, a pair of DNA oligos with 20 bp targeting sequences and appropriate 4 nt overhangs were synthesized (Sangon Biotech, China) and then annealed to the dsDNA duplex. Then, the dsDNA oligos were inserted into the BsaI sites of the pRGEB32B0, B3, and B5 vector, respectively. These Cas9/gRNA plasmid constructs were subsequently transformed into rice calli (ToWin Biotech, Wuhan, China). The DNA oligos used for gRNA cloning are listed in Table S3.

### Genomic DNA extraction and genotyping of gene-edited lines

The genomic DNA of the rice leaves was extracted using the cetyltrimethylammonium bromide method (Chen et al. 2022). To examine the genotypes of the targeted genes, the gene fragments flanking the editing sites were amplified using 2× Taq PCR MasterMix (Aidlab, China). Then, the PCR products were analyzed via Sanger sequencing. The primer sequences are listed in Table S3.

### Rice blast inoculation

The *Magnaporthe oryzae* isolate 99-20-2 (Zhou et al. 2018) was used to infect rice leaves as described previously (Chen et al. 2022). Briefly, the fully expanded 3rd leaf was detached from rice plants and inoculated with 2 μL of blast spores (3 × 10^5^ spores/mL) via a pipette tip. At 6 days postinoculation, the lesion area was calculated using ImageJ (imagej.nih.gov/ij/). The relative fungal growth in the inoculated leaves was determined by quantitative real-time PCR using the *M. oryzae* 28S rDNA and rice 25S rDNA (Xie et al. 2014) (Table S3).

### RNA extraction and RT–qPCR

Total RNA was extracted using TRIzol reagent (Thermo Fisher Scientific). For cDNA synthesis, 2 μg of total RNA was treated with DNase I (New England Biolabs) before reverse transcription. First-strand cDNA was synthesized using reverse transcriptase M-MLV (RNase H-) (Takara Bio) with Oligo(dT)_18_. Quantitative PCR was performed on a QuantStudio 3 (Applied Biosystems) with a One Step TB Green PrimeScript RT–PCR Kit (Takara Bio). Finally, the relative expression of the analyzed genes was calculated using the 2^−ΔΔCt^ method with rice *UBIQUITIN10* as the internal reference gene (Li et al. 2022).

### Supplementary Information

Below is the link to the electronic supplementary material.

Supplementary file1 (DOCX 31 kb)

## Data Availability

All data supporting the findings of this study are available within the paper and supplementary information.
