# Supplementary material for: C-terminal frameshift mutations generate viable knockout mutants with developmental defects for three essential protein kinases
Source: aBIOTECH. 2024 May 15;5(2):219–24. doi: 10.1007/s42994-024-00165-5 (PMC11224195; doi:10.1007/s42994-024-00165-5)
Supplement: Supplementary file 1 — Supplementary file1 (DOCX 31 kb) [file 42994_2024_165_MOESM1_ESM.docx]

# C-terminal frameshift mutations generate viable knockout mutants with developmental defects for three essential protein kinases

Yun Zhang^1, 2^, Miao-Miao Cui ^2^, Run-Nan Ke^1, 2^, Yue-Dan Chen^1, 2^, Kabin Xie^1, 2,^ *

^1^National Key Laboratory of Crop Genetic Improvement, Hubei Hongshan Laboratory, Huazhong Agricultural University, Wuhan 430070, China

^2^Hubei Key Laboratory of Plant Pathology, Huazhong Agricultural University, Wuhan 430070, China

* Correspondence (email: [kabinxie@mail.hzau.edu.cn](mailto:kabinxie@mail.hzau.edu.cn))

**Supplementary Information**

**Table of contents**

[Table S1. The genotype of the OsMPK1 CRISPR line (PTG147) in the T_0_ generation 3](#_Toc162280787)

[Table S2. The genotypes of *R942* and *R753* CRISPR-T_0_ plants 5](#_Toc162280788)

[Table S3. DNA oligos used in this study. 6](#_Toc162280789)

Table S1 The genotype of the OsMPK1 CRISPR line (PTG147) in the T0 generation

| **Lines** | **Genotype** | | **Number of seeds** |
| --- | --- | --- | --- |
| WT(NC) | GACTTCGAGCAGCATGCATTGTCCGAGGAAC | | 378 |
| PTG147-1 | Allele1(-4) | GACTTCGAGCAGCATGCA---- CCGAGGAAC | 0 |
|  | Allele2(+1) | GACTTCGAGCAGCATGCATTGTTCCGAGGAAC |  |
| PTG147-2 | Allele1(WT) | GACTTCGAGCAGCATGCATTGTCCGAGGAAC | 172 |
|  | Allele2(+1) | GACTTCGAGCAGCATGCATTGTTCCGAGGAAC |  |
| PTG147-3 | Allele1(-1) | GACTTCGAGCAGCATGCATT- TCCGAGGAAC | 102 |
|  | Allele2(-18) | GACT- - - - - - - - - - - - - - - - - - CCGAGGAAC |  |
| PTG147-4 | Allele1(-2) | GACTTCGAGCAGCATGCATT - - CCGAGGAAC | 0 |
|  | Allele2(-1) | GACTTCGAGCAGCATGCATT - TCCGAGGAAC |  |
| PTG147-5 | Allele1(-18) | GACT- - - - - - - - - - - - - - - - - - CCGAGGAAC | 110 |
|  | Allele2(-2) | GACTTCGAGCAGCATGCATT - - CCGAGGAAC |  |
| PTG147-6 | Allele1(-141) | GACTTCGAGCAGCATGCATT - - - 141 bp - - - GAC | 189 |
|  | Allele2(+18-16) | GACTTCGAGCAGCAGCTGGTGTTCTAATCTAC- 16 bp - C |  |
| PTG147-7 | Allele1(-4) | GACTTCGAGCAGCATGCA - - - - CCGAGGAAC | 3 |
|  | Allele2(-7) | GACTTCGAGCAGCAT - - - - - - - CCGAGGAAC |  |
| PTG147-8 | Allele1(-2) | GACTTCGAGCAGCATGCATT - - CCGAGGAAC | 0 |
|  | Allele2(+1) | GACTTCGAGCAGCATGCATTGTTCCGAGGAAC |  |
| PTG147-9 | Allele1(-1) | GACTTCGAGCAGCATGCATTG-CC GAGGAAC | 0 |
|  | Allele2(+1) | GACTTCGAGCAGCATGCATTGTCCCGAGGAAC |  |
| PTG147-12 | Allele1(-1) | GACTTCGAGCAGCATGCATT- TCCGAGGAAC | 107 |
|  | Allele2(-3) | GACTTCGAGCAGCATGCAT - - - CCGAGGAAC |  |
| PTG147-13 | Allele1(-1) | GACTTCGAGCAGCATGCATT-TCCGAGGAAC | 491 |
|  | Allele2(G>A) | GACTTCGAGCAGCATGCATTATCCGAGGAAC |  |
| PTG147-15 | Allele1(-1) | GACTTCGAGCAGCATGCATT- TCCGAGGAAC | 0 |
|  | Allele2(+1) | GACTTCGAGCAGCATGCATTGTTCCGAGGAAC |  |
| PTG147-16 | Allele1(-1) | GACTTCGAGCAGCATGCATT- TCCGAGGAAC | 0 |
|  | Allele2(+1) | GACTTCGAGCAGCATGCATTGTTCCGAGGAAC |  |
| PTG147-17 | Allele1(-2) | GACTTCGAGCAGCATGCAT--TCCGAGGAAC | 0 |
|  | Allele2(-2) | GACTTCGAGCAGCATGCAT--TCCGAGGAAC |  |
| PTG147-18 | Allele1(-104) | - - - - - - - - - - - - - 104 bp - - - - - - - - - - - - - - - - - -C | 0 |
|  | Allele2(-1) | GACTTCGAGCAGCATGCATT - TCCGAGGAAC |  |
| PTG147-19 | Allele1(-6) | GACTTCGAGCAGCATGC - - - - - - CGAGGAAC | 676 |
|  | Allele2(-1) | GACTTCGAGCAGCATGCATT - TCCGAGGAAC |  |
| PTG147-21 | Allele1(-2) | GACTTCGAGCAGCATGCATT - - CCGAGGAAC | 0 |
|  | Allele2(+1) | GACTTCGAGCAGCATGCATTGTTCCGAGGAAC |  |
| PTG147-22 | Allele1(-4) | GACTTCGAGCAGCATGC - - - - TCCGAGGAAC | 2 |
|  | Allele2(-59) | GACTTCGAGCAGCATGCATT - - - - - - - - - 59 bp |  |

The targeting sequences and PAM are highlighted with a yellow background and blue color, respectively. -, deletions; red characters, substitutions/insertions. NC, negative transformants were used as control.

Table S2 The genotypes of *R942* and *R753* CRISPR-T_0_ plants

| **T_0_ Lines** | **genotype** | | **Number of seeds** | |
| --- | --- | --- | --- | --- |
| ***R942* (OsWAK72)----LOC_Os07g31130** | | | | |
| WT(NC) | Allele1 | TCCGAGG TGACGAAGAGACCATCCATG | | 254 |
|  | Allele2 | TCCGAGG TGACGAAGAGACCATCCATG | |  |
| R942-81 | Allele1(WT) | TCCGAGG TGACGAAGAGACCATCCATG | | 115 |
|  | Allele2(+1) | TCCGAGGATGACGAAGAGACCATCCATG | |  |
| R942-87 | Allele1(+1) | TCCGAGGATGACGAAGAGACCATCCATG | | 5 |
|  | Allele2(-10) | TCCGAGG - - - - - - - - - - ACCATCCATG | |  |
| ***R753* (LRR-RLK)----LOC_Os01g13800** | | | | |
| WT(NC) | Allele1 | AAGCTGTCGCCATACTACTACGAGGAC | 150 | |
|  | Allele2 | AAGCTGTCGCCATACTACTACGAGGAC |  |  |
| R753-32 | Allele1(-1) | AAGCTGTCGCCATACTACTAC-ACGAGGAC | 0 | |
|  | Allele2(+1) | AAGCTGTCGCCATACTACTTACGAGGAC |  |  |
| R753-42 | Allele1(+1) | AAGCTGTCGCCATACTACTTACGAGGAC | 8 | |
|  | Allele2(+1) | AAGCTGTCGCCATACTACTTACGAGGAC |  |  |
| R753-77 | Allele1(+1) | AAGCTGTCGCCATACTACTTACGAGGAC | 20 | |
|  | Allele2(+1,-5) | AAGCTGTCGCCATAA- - - - - ACGAGGAC |  |  |
| R753-84 | Allele1(+1) | AAGCTGTCGCCATACTACTTACGAGGAC | 15 | |
|  | Allele2(+1,-5) | AAGCTGTCGCCATAA- - - - - ACGAGGAC |  |  |

The targeting sequences and PAM are highlighted with a yellow background and blue color, respectively. -, deletions; red characters, substitutions/insertions. NC, negative transformants were used as control.

Table S3 DNA oligos used in this study.

| **Genes**  **(RAP-DB ID)** | **Primer Name** | **Primer sequence (5’->3’)** |
| --- | --- | --- |
| **Primers for genotyping** | | |
| *OsMPK1* Os06g0154500 | OsMPK1-F | CCTAATCCAGGTGGTATAGGTGTG |
|  | OsMPK1-R | CAGCTACCCAGGAACTTGATCAC |
| *R942*  Os07g0493200 | R942-F | CGCTTGTAACCAGTCATGGGA |
|  | R942-R | GGCGCTTGCATTATAGCATCTACTAC |
| *R753*  Os01g0239700 | R753-F | CCGCCAGTCGATCCAGAGTT |
|  | R753-R | ACAGCTCCAGAGTTAACCGTG |
| **Primers for qPCR** | | |
| *OsUBQ10*  Os02g0161900 | OsUBQ10-qF | TGGTCAGTAATCAGCCAGTTTG |
|  | OsUBQ10-qR | CAAATACTTGACGAACAGAGGC |
| *OsMPK1* Os06g0154500 | OsMPK1-qF | AGCCATGGCGGGAGGTT |
|  | OsMPK1-qR | CGGTGACCTCGAACACGTT |
| *OsPR5*  Os12g0628600 | OsPR5-qF | TACAACGTCGCCATGAGCTTCT |
|  | OsPR5-qR | TGGGCAGAAGACGACTTGGTAGTT |
| M.grisea 28S rDNA | M.grisea28S rDNA-F | TACGAGAGGAACCGCTCATTCAGATAATTA |
|  | M.grisea28S rDNA-R | TCAGCAGATCGTAACGATAAAGCTACTC |
| 25S rDNA | 25S ribosomal RNA-F | AAGGCCGAAGAGGAGAAAGGT |
|  | 25S ribosomal RNA-R | CGTCCCTTAGGATCGGCTTAC |
| **Primers for Cas9 constructs** | | |
| OsMPK1-KO | p32B-MPK1-F | TGCACGAGCAGCATGCATTGTCCG |
|  | p32B-MPK1-R | AAACCGGACAATGCATGCTGCTCG |
| R942-KO | p32B-R942-F | TGCAGGATGGTCTCTTCGTCACCT |
|  | p32B-R942-R | AAACAGGTGACGAAGAGACCATCC |
| R753-KO | p32B-R753-F | TGCAGCTGTCGCCATACTACTACG |
|  | p32B-R753-R | AAACCGTAGTAGTATGGCGACAGC |
